# Characterization of *In Vivo* Retinal Lesions of Diabetic Retinopathy Using Adaptive Optics Scanning Laser Ophthalmoscopy

**DOI:** 10.1155/2018/7492946

**Published:** 2018-05-02

**Authors:** Sonja G. Karst, Jan Lammer, Salma H. Radwan, Hanna Kwak, Paolo S. Silva, Stephen A. Burns, Lloyd Paul Aiello, Jennifer K. Sun

**Affiliations:** ^1^Beetham Eye Institute, Joslin Diabetes Center, Boston, MA, USA; ^2^Department of Ophthalmology and Optometry, Medical University of Vienna, Vienna, Austria; ^3^Department of Ophthalmology, Cairo University, Cairo, Egypt; ^4^Department of Ophthalmology, Harvard Medical School, Boston, MA, USA; ^5^School of Optometry, Indiana University, Bloomington, IN, USA

## Abstract

**Purpose:**

To characterize hallmark diabetic retinopathy (DR) lesions utilizing adaptive optics scanning laser ophthalmoscopy (AOSLO) and to compare AOSLO findings with those on standard imaging techniques.

**Methods:**

Cross-sectional study including 35 eyes of 34 study participants. AOSLO confocal and multiply scattered light (MSL) imaging were performed in eyes with DR. Color fundus photographs (CF), infrared images of the macula (Spectralis, Heidelberg), and Spectralis spectral domain optical coherence tomography SDOCT B-scans of each lesion were obtained and registered to corresponding AOSLO images.

**Main Outcome Measures:**

Individual lesion characterization by AOSLO imaging. AOSLO appearance was compared with CF and SDOCT imaging.

**Results:**

Characterized lesions encompassed 52 microaneurysms (MA), 20 intraretinal microvascular abnormalities (IRMA), 7 neovascularization (NV), 11 hard exudates (HE), 5 dot/blot hemorrhages (HEM), 4 cotton wool spots (CWS), and 14 intraretinal cysts. AOSLO allowed assessment of perfusion in vascular lesions and enabled the identification of vascular lesions that could not be visualized on CF or SDOCT.

**Conclusions:**

AOSLO imaging provides detailed, noninvasive *in vivo* visualization of DR lesions enhancing the assessment of morphological characteristics. These unique AOSLO attributes may enable new insights into the pathological changes of DR in response to disease onset, development, regression, and response to therapy.

## 1. Introduction

Diabetic retinopathy (DR) is characterized by hallmark retinal lesions including microaneurysms (MA), hard exudates (HE), cotton wool spots (CWS), intraretinal hemorrhages, and retinal neovascularization (NV) which are present in over 77–90% of individuals after 15 or more years of diabetes [1–3]. The distribution and extent of these lesions determine DR severity and predict the risk of worsening DR [4–8]. Thus, crucial clinical management decisions including recommendations for follow-up and treatment are dependent on the ability to accurately assess DR lesions over time.

Color fundus photography is the standard method by which DR severity is assessed for clinical and research purposes [1]. Alternative imaging methods such as spectral domain optical coherence tomography (SDOCT), scanning laser ophthalmoscopy (SLO), and fluorescein angiography (FA) allow evaluation of specific aspects of retinal pathology such as neural retinal layer thickening, disorganization or disruption, and vascular leakage, respectively, and have also been utilized to assess individual DR lesions in detail [9–14]. However, all these imaging modalities are limited by a lateral resolution of approximately 10–15 *μ*m and are thus unable to resolve structural details at the cellular level. Although there are numerous histological studies of DR lesions at the cellular level in human postmortem tissues, similarly detailed *in vivo* evaluation has been limited [15–17].

The adaptive optics (AO) systems allow ultrahigh resolution assessment of the human retina *in vivo* [18–23]. AO technology compensates for ocular wave front errors primarily induced by the cornea and lens and allows correction of >90% of the optical aberrations within an individual eye, thus providing a theoretical lateral resolution limit of 1.4 *μ*m for large pupils and short wavelength light [24]. AOSLO can also capture video output, allowing dynamic visualization of intravascular blood cell flow in vessels down to the capillary level [25–28].

In this study, we characterized vascular and nonvascular hallmark DR lesions using a custom-built AOSLO with a lateral resolution of approximately 2.5 *μ*m. Whereas previous reports have focused on the overall capillary network, MA, and intraretinal microvascular abnormalities in the diabetic eye, this manuscript also evaluates confocal and multiply scattered light imaging findings for the following additional diabetic lesions that have not been systematically assessed using AOSLO: retinal neovascularization, hard exudates, hemorrhages, cotton wool spots, and intraretinal cysts [28–32]. We document the longitudinal history of selected individual lesions as well as the response of particular lesions to antivascular endothelial growth factor (VEGF) therapy. In addition, we systematically describe the AOSLO characteristics in static and dynamic (video) assessments for each lesion type recorded with two AOSLO acquisition modes: confocal imaging and aperture offset imaging. Advantages and disadvantages of the AOSLO technique in relation to traditional color fundus photography and SDOCT are also presented.

## 2. Methods

The study was approved by the Institutional Review Board of the Joslin Diabetes Center, and all study procedures adhered to the tenets of the Declaration of Helsinki. Prior to study inclusion, informed consent was obtained from all subjects.

Subjects were eligible for the study if they met the following inclusion criteria: age 18 years or older, diagnosis of type 1 or type 2 diabetes mellitus as defined by the American Diabetes Association, optical media clear enough to obtain good quality images, and stable central fixation [33]. Participants with substantial macular pathology attributable to nondiabetic eye disease, such as age-related macular degeneration, retinal vein occlusion, uveitis, and Irvine Gass syndrome were excluded from participation.

All participants received a comprehensive dilated ophthalmologic examination followed by retinal imaging including SDOCT (Spectralis Heidelberg Engineering, Germany), ETDRS 7 standard field color stereoscopic fundus photography (Carl Zeiss Meditec Inc., Dublin, CA) or ultrawide field retinal imaging (Optos PLC, Scotland, United Kingdom), and AOSLO (Boston Micromachines Corp., Cambridge, MA). AOSLO imaging and SDOCT imaging were performed at each visit on the same day. For SDOCT imaging, cubic (20° × 20° field, 49 B-scans, 16 frames ART mean, and high resolution setting) and detailed (15°× 5° field, 24 B-scans, 25 frames ART mean, and high resolution setting) macular volume scan patterns centered on the fovea were performed. Details of the AOSLO imaging are provided below. The axial length of each study eye was determined using an IOL Master (Carl Zeiss Meditec, Dublin, CA) in order to subsequently convert angular to metric coordinates on the AOSLO images.

**Figure 1:**
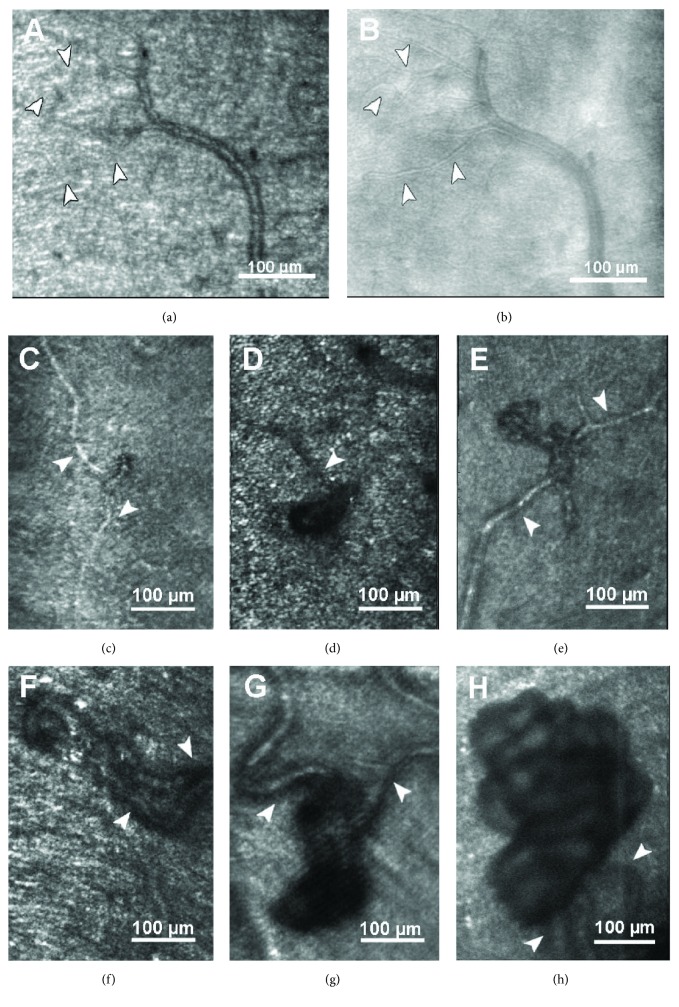
(a) Example of an arteriole in confocal and multiply scattered light (MSL) and (b) AOSLO imaging. Finer details of the vessel walls are visualized with MSL AOSLO imaging, which increases the sensitivity to scattered light reflected from vasculature and erythrocytes. (c–f) Intraretinal microvascular abnormalities (IRMA). (g, h) Neovascular proliferations (NVE) growing anteriorly to the RNFL. Arrowheads indicate feeder/draining vessels. In (d), the AOSLO image is focused at the photoreceptor level and displays a regular photoreceptor mosaic surrounding the blurred shadow of an IRMA. In (g) and (h), the 3-dimensional structure of the NVE results in clear focus on some vascular loops and blurry vessel formations in other areas of the NVE.

The AOSLO used in this study was a double pass, single deformable mirror version of the Indiana system that has been previously described [34, 35]. AOSLO images were acquired confocally, using a standardized protocol that obtained images focused at the following planes: the lesion of interest, posteriorly at the photoreceptor level and anteriorly at the nerve fiber layer. In addition, a multiply scattered light (MSL) or pinhole aperture offset technique as recently described was utilized to image each DR lesion (Figures 1(a) and 1(b)) [36, 37]. With this technique, images are generated from spatial variations of multiply scattered light leading to higher contrast, especially of vessel walls and erythrocytes, as the specular component of the image is reduced by the offset aperture [38]. Aperture size and displacement (ranging from 25 *μ*m to 500 *μ*m for size and 0 *μ*m to 350 *μ*m for displacement) were adjusted for individual lesions and eyes to obtain the best quality image possible and depth of focus ranged between 80 *μ*m and 150 *μ*m. However, a 500 *μ*m aperture which was displaced by 300 *μ*m (~5 Airy disk diameters) perpendicular to the targeted lesion was used to acquire most images. The displacement was adjusted using a computer-controlled, motorized positioning stage allowing a positioning accuracy of approximately 1 *μ*m. AOSLO image acquisition sessions ranged between 15 and 60 minutes depending on the number of lesions and size of area scanned.

**Figure 2:**
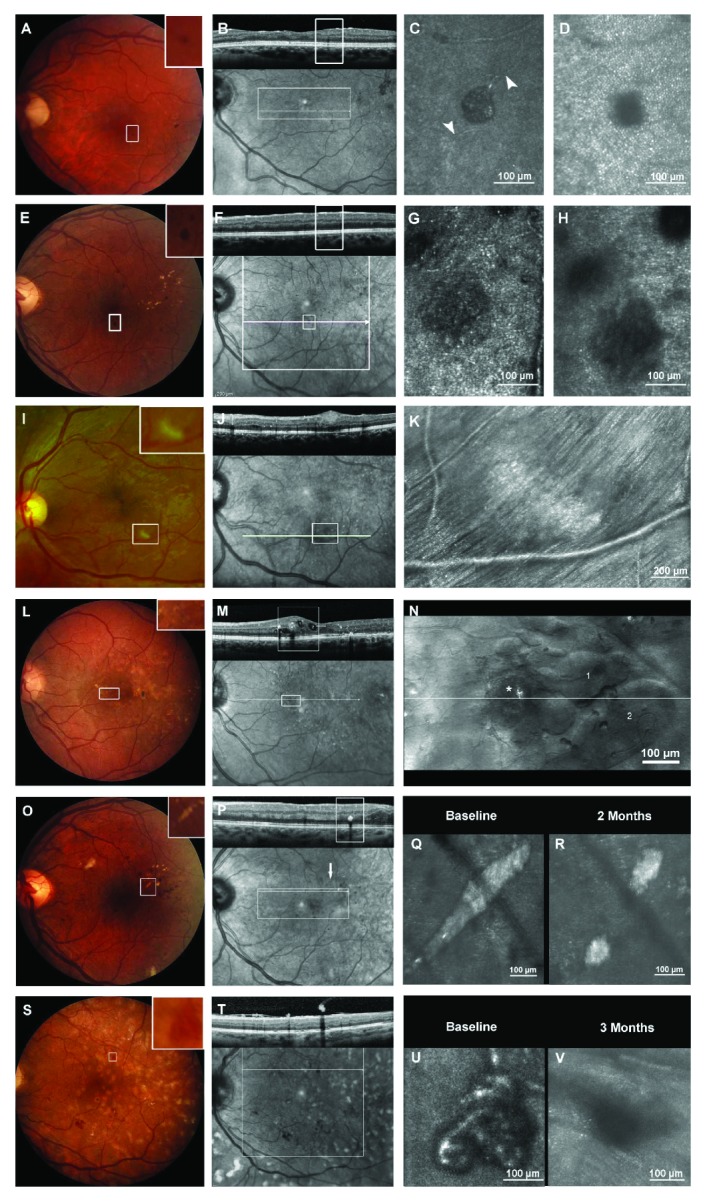
Multimodal imaging (color fundus photo, OCT, IR image, and AOSLO) of six hallmark lesions: a microaneurysm, MA (A–D), a blot hemorrhage (E–H), a cotton wool spot, CWS (I–K), intraretinal cysts (L–N), hard exudate, HE (O–R), and neovascular proliferation, NV (S–V). (A) Microaneurysm visible as small red dot in 30° color fundus photo (CF) centered on the macula. (B) In the corresponding B-scan the inner plexiform layer, MA is visible primarily by its shadowing effect on the outer retinal layers. (C) In the AOSLO image focused at the vascular level, the MA is visible as a well-defined saccular bulge within the capillary network. Feeding and draining vessels (arrowheads) are discernable. (D) When focused at the photoreceptor plane, shadowing from the MA is present surrounded by a clearly imaged photoreceptor mosaic. (E) Small blot hemorrhage easily visible in 30° color fundus photo and Spectralis IR image (F) but not clearly identifiable in the corresponding cross-sectional SDOCT B-scan. G Outlines of the hemorrhage are blurred even in the AOSLO image focused at the hemorrhage plane. (H) When focused on the photoreceptor plane, there is shadowing of the photoreceptor mosaic by the anteriorly located blot hemorrhage. (I) CWS clearly visible in 30° Optos color fundus image and Spectralis IR image (J). In the corresponding SDOCT B-scan, the CWS appears as a nodular thickening of the retinal nerve fiber layer (RNFL) compressing the inner plexiform and inner nuclear layer. Notice the faint shadowing effect on the outer retinal layers. (K) Montage of 17 AOSLO images focused at the RNFL level to cover an area of 3° × 4.5°. The CWS appears hyperreflective with a less clearly delineated nerve fiber striation pattern compared to adjacent areas. Nerve fiber bundles adjacent to the CWS are pushed aside at the lower right border of the lesion. (L) DME is not evident on the 30° color fundus and Heidelberg Spectralis IR (M) photographs, but is clearly visible on the montaged MSL AOSLO images (N) and corresponding SDOCT B-scan. The white boxes in (L) and (M) correspond to the area imaged on AOSLO (N). An asterisk marks a microaneurysm and [1, 2] indicates corresponding cysts in the AOSLO (N) and SDOCT (M) images. (O) The HE is clearly visible on color fundus photography (O) OCT and IR image (P). In confocal AOSLO images (Q), the HE appears as a hyperreflective granular structure with an internal honeycomb-like pattern. Two months after intravitreal ranibizumab injections for center involved diabetic macular edema, the HE decreased in size to reveal an intact photoreceptor pattern. (S) Fundus photograph of neovascularization elsewhere (NVE) that is clearly visible on OCT and IR image (T). (U) Corresponding NVE imaged with AOSLO before treatment (U) and 3 months after administration of intravitreal ranibizumab (V). AOSLO imaging reveals the persistence of an involuted neovascular formation.

Image processing was performed using a customized Matlab platform (MatLab, The MathWorks, Natick, MA) and took approximately 30 min per image. Sinusoidal distortion artifacts were corrected utilizing a polynomial dewarping algorithm [35]. After manual selection of 5–50 frames from each video block, automated image alignment and averaging were performed. Individual diabetic retinal lesions were characterized by size, shape, and appearance from the averaged images, and perfusion status was assessed on AOSLO videos viewed in ImageJ (NIH, Bethesda, Maryland). Individual lesions were registered to widefield SDOCT IR images or color fundus photographs by manually or semi automatically montaging 3–20 adjacent AOSLO images and then identifying comparable vessel landmarks on each image set with guidance from recorded AOSLO navigation coordinates. Once registration was completed, corresponding SDOCT B-scans were assessed in order to determine key features of each lesion's appearance on SDOCT such as visibility, presence of associated hyperreflectivity, and location within or anterior to the neural retinal layers.  Figure 2 contains examples of 6 lesions obtained using the different imaging modalities in this study: color and IR photographs, SDOCT B-scans, and AOSLO images.

## 3. Results

DR lesions were imaged in 35 eyes of 34 participants (16 females, mean age 41 ± 12.5 years). The mean duration of DM was 24 ± 8 years (28 type 1 DM), and mean HbA1c was 8 ± 2%. DR severity grading of the 35 eyes that were included was as follows: 2 mild, 9 moderate, 8 severe nonproliferative DR, and 16 proliferative DR.

The lesions that were evaluated included microaneurysms (MA, *N* = 52), intraretinal microvascular abnormalities (IRMA, *N* = 20), retinal neovascularization (NV, *N* = 7), hemorrhages (HEM *N* = 5), hard exudates (HE, *N* = 11), cotton wool spots (CWS, *N* = 4), and intraretinal cysts (*N* = 14) (Table 1). In the following sections, the main AOSLO characteristics are systematically described. A more detailed description observed for each lesion type using static confocal imaging, MSL imaging, and dynamic (video) assessment as well as a thorough comparison to other imaging modalities is available at the journal's website.

### 3.1. Vascular Lesions

**Figure 3:**
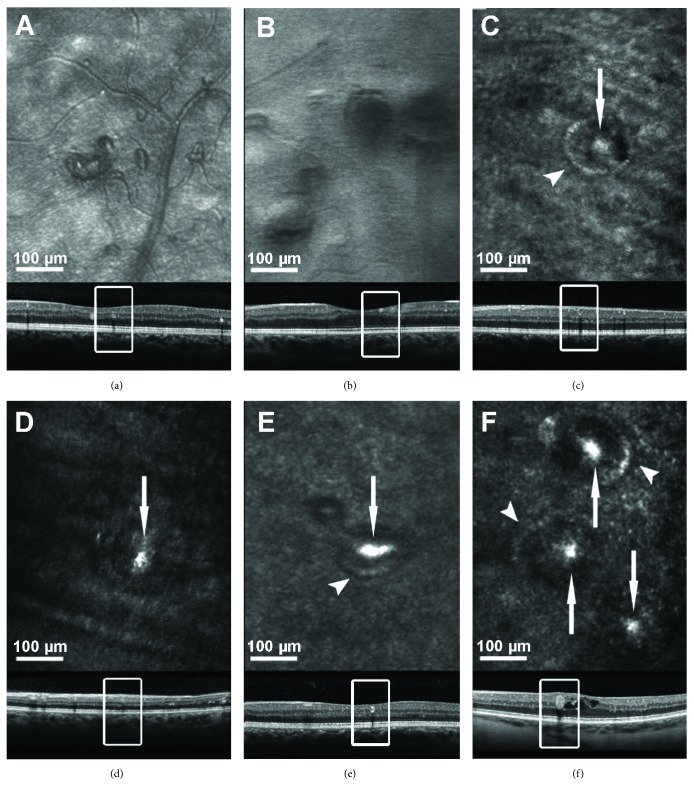
Six examples of microaneurysms (MA) on AOSLO. The rectangles indicate the MA location in the corresponding SDOCT B-scans. Some MAs have hyperreflective vessel walls (arrowhead) or intraluminal hyperreflective structures (arrow) in AOSLO images (c–f). Using MSL AOSLO imaging (a, b), MA vessel walls are more clearly visible compared to confocal AOSLO imaging (c–f).

Small vascular lesions like MA, IRMA, and NV that were sometimes hard to detect or distinguish in IR images or fundus photos could be clearly identified in AOSLO images. In confocal images, vessel walls of these vascular lesions were markedly thickened and appeared darker compared to vessel walls of normal intraretinal capillaries. In some MA, focal areas of granular hyperreflectivity were present along their wall in 35% (*n* = 18) or within their lumen (46% *n* = 24) (Figures 3(c)–3(f)). Wall hyperreflectivity present on AOSLO imaging was not always present in SDOCT images (31%) and vice versa (59%). SDOCT intraluminal hyperreflectivity was observed in 17 MA (57%). Only 9 of these 17 MA (53%) showed intraluminal hyperreflectivity in corresponding AOSLO images.

**Figure 4:**
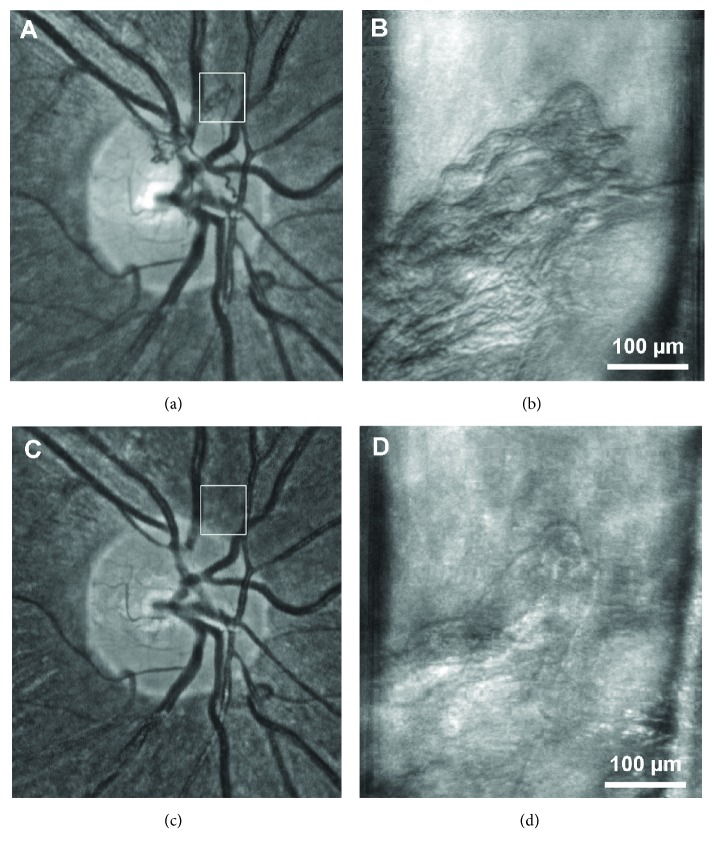
Response of proliferative diabetic retinopathy to anti-VEGF therapy on AOSLO. (a) Fundus photograph of neovascularization at the optic disc (NVD). (b) MSL AOSLO image of the area highlighted by the white box in (a). (c, d) Same NVD 4 weeks after administration of intravitreal ranibizumab. The NVD can no longer be identified in the infrared image; however, AOSLO imaging reveals the persistence of an involuted neovascular formation.

MSL imaging technique revealed more sharply defined vessel walls than confocal imaging, so perfused and nonperfused vascular channels could be clearly identified (Figures 1(a) and 1(b)). This distinction was particularly evident in imaging areas of fibrosis within patches of NV (Figure 4). These structural findings were complemented by dynamic assessment, as blood cell flow was clearly visible in all perfused vascular lesions. Though blood flow could not be quantified, it appeared qualitatively slower (often markedly so) in some MA or regressing neovascular tissue, particularly in the eyes that had undergone treatment with antivascular endothelial growth factor (VEGF) therapy or panretinal photocoagulation (Videos 5 and 6).

### 3.2. Nonvascular Lesions

Hemorrhages presented with distinct border and homogenously hyporeflective internal appearance. Although HEM could not be differentiated from MA in color fundus photos or IR images, they could be easily distinguished from perfused MAs on AOSLO due to the hemorrhage's lack of blood flow, hyperreflective foci, and/or adjacent feeder vessels. Hemorrhages were not visible in SDOCT.

**Figure 5:**
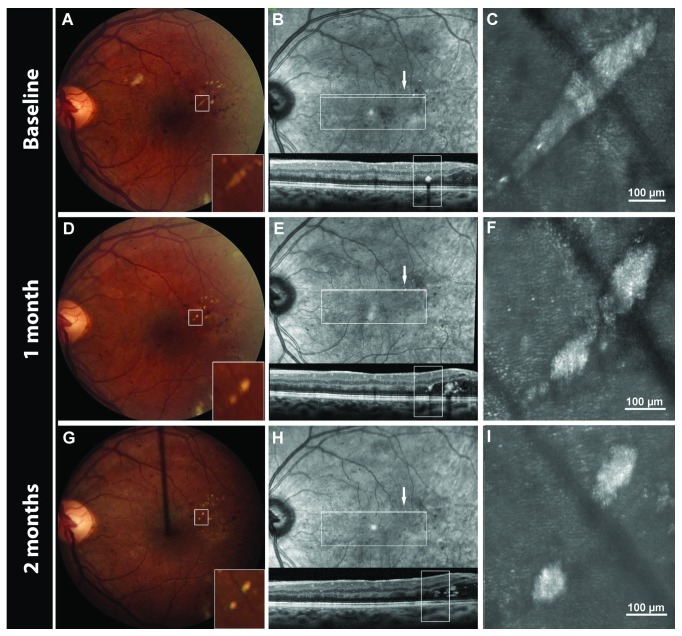
Multimodal imaging of a resolving hard exudate (HE) over a 2-month period. Intravitreal ranibizumab injections for center-involved diabetic macular edema were performed at the baseline and 1-month visits. The HE is clearly visible on color fundus photography (A, D, G) and IR images (B, E, H). Its location within the outer nuclear layer can be determined in cross-sectional SDOCT B-scans (B, E, H). Confocal AOSLO images (C, F, I) of the HE.

Hard exudates were visible in SDOCT, IR images, and color fundus photography. In confocal AOSLO images, HE appeared as irregularly shaped, grainy-appearing hyperreflective patches with dark borders (Figure 2, Q) casting a shadow on the photoreceptor mosaic. With high resolution AOSLO imaging, changes in HE size after anti-VEGF treatment could be measured more accurately than on standard color fundus photography or on SDOCT B-scans (Figure 5). In addition, AOSLO imaging showed that the underlying photoreceptor mosaic remained intact and was gradually revealed as the HE were resolved (Figure 2, R).

Cotton wool spots were visible in all imaging modalities applied whereas confocal AOSLO images revealed more details than MSL images. In confocal AOSLO, CWS appeared hyperreflective in comparison to the surrounding retinal tissue (Figure 2, K). Within each CWS, the RNFL striation pattern was less distinct and boundaries between the RNFL bundles could not always be clearly identified. Individual RNFL bundles within each CWS were wider in diameter than the nerve fiber bundles outside but immediately adjacent to the CWS, which appeared compressed and displaced at the border of the lesions.

Intraretinal cysts were difficult to visualize on fundus photographs or IR images but were clearly visualized on SDOCT images. Though intraretinal cysts could not be identified in confocal AOSLO images, the MSL imaging technique allowed clear delineation of cyst boundaries (Figure 2, N) and wall structures. Lateral cyst dimensions and proximity to different retinal structures could be well defined due to the ability to precisely discern cyst wall boundaries.

## 4. Discussion

This study provides the first detailed, systematic description of multiple vascular and nonvascular retinal lesions of diabetic retinopathy as imaged using noninvasive confocal and multiply scattered light AOSLO technology. In comparison to the previous studies of AOSLO which have evaluated either more global features of the diabetic capillary network or limited their focus to specific vascular lesions such as MAs, this investigation provides a broad survey that directly compares the appearance of a diverse set of diabetic pathologies on AOSLO imaging to that on standard fundus photography and SDOCT scans [30–32, 37].

Small lesions of clinical importance, including neovascularization and microaneurysms, were readily detectable on AOSLO even when they were not visualized using SDOCT or standard color fundus photographs. AOSLO also allowed longitudinal monitoring of structural changes at the cellular level over time, including retinal anatomic response following therapeutic intervention. Vascular perfusion was often detectable with AOSLO even when the lesion itself was undetectable by other imaging modalities or when a vascular lesion appeared entirely fibrotic and nonperfused on standard retinal photographs. Thus, AOSLO promises earlier detection and more precise determination of structural changes in the diabetic eye both over time and in response to treatment than that currently available with other standard imaging modalities. The high sensitivity of AOSLO to detect intraretinal lesions such as MAs and hemorrhages could be a reason for altered photoreceptor counts in diabetic patients due to shadowing artifacts from early diabetic lesions in subclinical DR [26].

A major advantage of AOSLO imaging is the ability to visualize intraluminal red blood cell flow in a detailed and dynamic fashion in combination with ultra-high resolution details of blood vessel walls. AOSLO videos can readily distinguish perfused MAs and NV from nonperfused lesions and can even distinguish areas of perfusion and nonperfusion within a single lesion. Although SDOCT and OCT angiography (OCTA) can localize NV location relative to the posterior hyaloid and retinal surface, blood flow assessment in OCTA is currently limited to certain blood flow velocities [14, 39, 40]. Areas of slow blood flow such as in MA or fibrotic NV may be missed. In contrary, red blood cell flow can be visualized independently of its velocity in AOSLO videos. A comparison of both imaging techniques was not within the scope of our study because OCTA images were not acquired in our patients. However, the ability to longitudinally evaluate perfusion changes of vascular DR lesions in the human eye may prove valuable in predicting the functional impact of antiangiogenic therapies on MAs, NV, and capillary occlusion [32].

The technique of multiply scattered light through decentration of the pinhole aperture, a recently introduced AOSLO imaging method, further enhances image quality of vessel walls and erythrocytes [36, 37]. Imaging of retinal vascular lesions including MAs, IRMA, and neovascularization is substantively improved by the use of this MSL technique due to improved visualization of vascular walls and individual blood cell flow. The MSL method also dramatically improves the ability to identify intraretinal cyst boundaries in the eyes with diabetic macular edema as compared with standard AOSLO confocal imaging. However, the MSL technique does not appear to offer substantial advantages over standard AOSLO confocal imaging in the evaluation of intraretinal hemorrhages, hard exudates, or cotton wool spots.

Hyperreflectivity on AOSLO images was observed in diverse lesion types and may have multiple etiologies. Hard exudates were brightly hyperreflective on AOSLO images, likely demonstrating high reflectance from lipid deposits. Cotton wool spots were also hyperreflective in comparison to the surrounding tissue, possibly resulting from edema of the nerve fiber layer and the accumulation of mitochondria, neurofilaments, and endoplasmic reticulum in enlarged axons [41, 42]. In addition, AOSLO hyperreflectivity was variably present within vascular walls and lumens of some MAs. Dubow et al. previously described intraluminal hypofluorescent areas imaged with AOSLO FA that might correspond to the static hyperreflectivity we observed in AOSLO images [30]. In both imaging methods, these intraluminal areas of different reflectivity indicate missing red blood cell flow which is consistent with intraluminal clotting. The histopathologic correlate of this AOSLO hyperreflectivity remains uncertain. Stitt et al. found degenerate lipid containing macrophages in some trypsin digest histologic preparations of MAs [43]. Clotting within MA lumens with basement membrane-like matrix and lipid-containing macrophages has also been described [43]. It is possible that AOSLO hyperreflectivity within MAs and along their walls might represent the presence of these lipid-containing macrophages.

Interestingly, hyperreflectivity of the MA wall on AOSLO images was not consistently correlated with the hyperreflective “ring sign” described by Horii et al. on SDOCT [44]. Similarly, AOSLO hyperreflectivity within MAs was not related to hyperreflectivity of the MA lumen on corresponding SDOCT images. These differences might arise from the different wavelengths used in these two imaging techniques or the use of a transverse cross-sectional scanning plane of SDOCT as opposed to the en-face scanning of AOSLO. Since individual SDOCT B-scans are discontinuous, there are gaps between each line scan that are not evaluated and individual lesions may not always be scanned perpendicularly. In contrast, with AOSLO en-face scanning, the axial scanning position is continuously variable in order to acquire a high level of detail covering the entire extent of the MA.

Hard exudates appear as irregularly shaped hyperreflective lesions independent of blood vessels on AOSLO imaging. Bolz et al. postulated that hyperreflective foci in SDOCT images of the eyes with DR are precursors of HE that accumulate in the OPL before becoming visible as HE in color fundus photographs [45]. The irregular granular structure of HEs in AOSLO is consistent with the hypothesis that HE consist of many small elements. However, small hyperreflective lesions consistent with HE precursors were not routinely identified on AOSLO images, perhaps due to interference from background reflectivity of the photoreceptors.

A general limitation of AOSLO imaging is that it can be difficult to determine the precise anteroposterior location of the image focal plane when this plane is located between the nerve fiber layer and photoreceptor layer. Thus, the anteroposterior extent of pathologies such as intraretinal cysts is better detected in cross-sectional SDOCT scans. Limitations specific to this study are the variable number of different intraretinal lesions imaged per eye which were selected in order to provide a broad range of pathology. We also did not compare AOSLO imaging to fluorescein angiography (FA) since AOSLO allows much higher resolution imaging in comparison to standard FA, and it is already well documented that AOSLO MSL imaging is similarly sensitive to AOSLO FA for the detection of perfused vasculature without the need for invasive use of contrast dyes. AOSLO MSL imaging also provides additional information about nonperfused vascular lesions and vascular wall structures that may not be evident on FA [27].

In summary, the ability to noninvasively visualize the hallmark retinal lesions of diabetic retinopathy in the human eye at a lateral resolution of 2.5 *μ*m using AOSLO has provided a level of characterization previously impossible. Longitudinal evaluation of individual lesions over time is now feasible, and with variable axial scanning, the three-dimensional characteristics of individual lesions become readily evident. Furthermore, utilizing high-resolution video sequences, AOSLO imaging enables assessment of individual blood cell flow within the retinal vasculature. These AOSLO attributes allow unprecedented evaluation of the retina and the pathologic changes induced by diabetes, potentially facilitating novel insights into the development, regression, and response to therapy of diabetic eye disease.

## Figures and Tables

**Table 1:** Diabetic retinopathy lesion characteristics on AOSLO and SDOCT imaging modalities.

Lesion type	Size range (*μ*m)	Appearance on AOSLO imaging		Increased reflectivity	Longitudinal follow-up	Shadowing of cones	Detectable in SDOCT (%)	Blood flow visible on AOSLO imaging
		Confocal	Multiply scattered light					
Microaneurysms (*n* = 52)	46–168	Round/oval lesions with dark, thickened vessel walls	Better defined vessel walls than in confocal imaging	46% intraluminal 35% vessel wall	No	Yes	58	Feeder vessels and intraluminal blood flow
IRMA (*n* = 20)	69–360	Distinct convoluted vessel formation	Better defined vessel walls than in confocal imaging	No	No	Yes	90	Feeder vessels and intraluminal blood flow
NV (*n* = 7)	283–1406	Distinct convoluted vessel formation	Sharply defined vessel walls, distinction to fibrotic tissue, perfused and nonperfused vessels	No	Yes, under treatment of 0.3 ranibizumab	Yes	100	Feeder vessels and intraluminal blood flow, nonperfused vascular channels
Hemorrhages (*n* = 5)	52–234	Dark homogenous patches	Dark homogenous patches, same information as in confocal imaging	No	No	Yes	No	n/a
CWS (*n* = 4)	432–954	RNFL striation pattern disrupted, hyperreflective, fluffy	Less RNFL reflectivity	Yes	No	No	100	n/a
HE (*n* = 11)	27–745	Highly reflective distinct granular patches	Highly reflective distinct granular patches, same information as in confocal imaging	Yes	Yes, under treatment of 0.3 ranibizumab	Yes	91	n/a
Cysts (*n* = 14)	72–1086	Blurred dark shadows	Clear delineation of cyst boundaries and wall structures	No	No	Inconsistent	100	n/a

AOSLO: adaptive optics scanning laser ophthalmoscopy; SDOCT: spectral domain optical coherence tomography; IRMA: intraretinal microvascular abnormalities; NV: neovascularization; CWS: cotton wool spot; RNFL: retinal nerve fiber layer; HE: hard exudates.

## Supplementary Materials

Supplementary 1 Video 1: example of an MA with perfusion visible throughout all portions of the lesion on confocal AOSLO imaging. Blood flow through the surrounding capillary network is also visible.

Supplementary 2 Video 2: a nonperfused MA imaged with the MSL technique. No blood flow is visible within the centrally located vascular lesion despite clearly visible flow through the surrounding vessels.

Supplementary 3 Video 3: the central, bean-shaped MA in this video demonstrates apparent deformability of the superior lesion wall with movement of blood cells adjacent to the wall. In contrast, the inferior wall of the MA is fixed and does not appear to be flexible in response to the passage of blood cells through the inferior portion of the MA.

Supplementary 4 Video 4: an example of a perfused IRMA on MSL imaging.

Supplementary 5 Video 5: video of retinal NV demonstrating adjacent vascular channels within the same area of NV that are perfused (located superiorly) and nonperfused (located inferiorly).

Supplementary 6 Video 6: area of retinal NV before (left panel) and after (right panel) treatment with intravitreal anti-VEGF therapy. After anti-VEGF treatment, many neovascular channels appear to have resolved entirely and others are substantially decreased in caliber. In contrast, the larger, nonneovascular vessels present along the left and bottom right edges of the videos do not appear to have changed noticeably in caliber or appearance.
